# Early Pregnancy Human Decidua is Enriched with Activated, Fully Differentiated and Pro-Inflammatory Gamma/Delta T Cells with Diverse TCR Repertoires

**DOI:** 10.3390/ijms20030687

**Published:** 2019-02-05

**Authors:** Antonia Terzieva, Violeta Dimitrova, Lyubomir Djerov, Petya Dimitrova, Silvina Zapryanova, Iana Hristova, Ivaylo Vangelov, Tanya Dimova

**Affiliations:** 1 Institute of Biology and Immunology of Reproduction, Bulgarian Academy of Sciences, 1113 Sofia, Bulgaria; anterzieva@abv.bg (A.T.); silvina_z@abv.bg (S.Z.); v_angel_off@abv.bg (I.V.); 2 Medical University, University Obstetrics and Gynecology Hospital “Maichin Dom”, 1431 Sofia, Bulgaria; dtdimitrova@mail.bg (V.D.); bubodjerov@abv.bg (L.D.); iana.hristova06@gmail.com (I.H.); 3 Institute of Microbiology, Bulgarian Academy of Sciences, 1113 Sofia, Bulgaria; petya_dimitrova@web.de

**Keywords:** gamma/delta T-cell effectors, decidua, TCR repertoire diversity, human pregnancy

## Abstract

Pregnancy is a state where high and stage-dependent plasticity of the maternal immune system is necessary in order to equilibrate between immunosuppression of harmful responses towards the fetus and ability to fight infections. TCR γδ cells have been implicated in the responses in infectious diseases, in the regulation of immune responses, and in tissue homeostasis and repair. The variety of functions makes γδ T cells a particularly interesting population during pregnancy. In this study, we investigated the proportion, phenotype and TCR γ and δ repertoires of γδ T cells at the maternal–fetal interface and in the blood of pregnant women using FACS, immunohistochemistry and spectratyping. We found an enrichment of activated and terminally differentiated pro-inflammatory γδ T-cell effectors with specific location in the human decidua during early pregnancy, while no significant changes in their counterparts in the blood of pregnant women were observed. Our spectratyping data revealed polyclonal CDR3 repertoires of the δ1, δ2 and δ3 chains and γ2, γ3, γ4 and γ5 chains and oligoclonal and highly restricted CDR3γ9 repertoire of γδ T cells in the decidua and blood of pregnant women. Early pregnancy induces recruitment of differentiated pro-inflammatory γδ T-cell effectors with diverse TCR repertoires at the maternal–fetal interface.

## 1. Introduction

Implantation, as a part of early pregnancy, is a dynamic process when the semi-allogeneic embryo “signals” its presence in the uterus and should be recognized and tolerated by the maternal immune system. At the same time, maternal immune cells must be capable of mounting some protective response against bacteria or pathogens that may harm the fetus. The type of implantation determines to what extent maternal immune cells interact with fetal tissues and cells. In interstitial (invasive) implantation and hemochorial type of placenta (human, rodent), there are two surfaces of contact—one maternal–fetal interface (MFI) is that wherein decidua basalis-based maternal immune cells contact with the extravillous cytotrophoblasts (EVTs), and another one, increasing in volume as pregnancy progresses, is formed between the chorionic villi and maternal blood, wherein the maternal immune cells in the peripheral circulation are in contact with syncytiotrophoblast, covering placental villi. It is now well accepted that a successful pregnancy requires a robust, dynamic and responsive maternal immune system and therefore the immunological milieu at the MFI is unique, modulated and definitely not suppressed [1]. The unique composition and phenotype of decidua-based immune cells as a part of decidua-associated lymphoid tissue (DALT) as well as their interaction with stromal decidual cells, and trophoblast (embryonic) cells is crucial for pregnancy recognition and establishment of tolerogenic environment for the embryo. In species with hemochorial placenta, leukocytes are about 10% of the decidual cells in the proliferative phase of the cycle, increasing to ~20% in the late secretory phase and >40% in early pregnancy [2].

Successful pregnancy is generally associated with downregulation of the adaptive conventional T-cell responses and compensatory overweight of the innate immune system, part of which are unconventional T lymphocytes—γδ T cells. Unlike the relatively well-studied decidual natural killer cells (dNK) which are the main innate population at the MFI during early pregnancy (70% of lymphocytes) [3], data about γδ T cells and their role in successful pregnancy are far from understood and quite controversial. γδ T cells are T lymphocytes using γ and δ chains for their T cell receptor, instead of α and β chains like conventional T cells do use [4]. Together with αβ T cells, they have been conserved for >450 million years of evolution [5]. There are two fundamental differences between αβ and γδ T lymphocytes: 1) in contrast to αβ T cells, γδ T cells do not (or only rarely) recognize peptides processed from complex protein antigens by professional antigen-presenting cells (APCs) but rather see unprocessed antigens such as phosphorylated microbial metabolites and lipid antigens; 2) their recognition mechanisms are major histocompatibility complex (MHC) unrestricted, which is not the case for conventional T cells [5,6]. As a rule, γδ T cells are cytotoxic and have anti-tumor and anti-microbial Th1 activity and share many of their receptors with NK cells, like activating and inhibitory NK receptors (NKRs) [6,7,8]. At the same time, γδ T cells are directly responsible for maintaining homeostasis and tissue repair in inflammation and metabolic stress conditions by recognizing stress-induced proteins and secretion of growth factors [9,10]. The number of γδ T cells in the blood and lymphoid tissues is relatively low (0.5–10%) because they reside mostly on the epithelial surfaces [5,6]. Like conventional αβ T cells and B cells, γδ T cells use V(D)J gene rearrangement to generate a set of highly diverse receptors to recognize antigens. This diversity is mainly generated in the complementarity-determining region 3 (CDR3), with an estimated repertoire of approximately 10^18^ possible combinations [4]. A great deal of the CDR3 region diversity is due to N nucleotide addition at the V-J and V-D-J junctions and multiple D segment rearrangements in the δ chain, which makes TCRγδ diversity even greater than that seen in αβ T cells [11,12]. The highly diverse repertoire of TCRγδ CDR3 sequences suggests that these regions can potentially recognize a vast array of distinct antigens.

In human, some authors reported that early pregnancy and peri-implantation are associated with accumulation of γδ T cells into the decidua [13], and that γδ T cells are in an increased amount in the 3rd trimester decidua, compared with the early decidua (1st trimester) [14]. The group of Bulmer JN did not find any change in the decidual γδ T-cell proportion, induced by pregnancy [15]. Series of studies have found that the reduction of peripheral γδ T lymphocytes is associated with pregnancy failure [16]. Conversely, other study has observed that women with pregnancy failure show an increased percentage of peripheral γδ Τ cells [17]. Although it has been accepted that γδ T cells are Th1 cells, it has been recently reported that on the basis of the microenvironment, γδ T cells can assume the features similar to those of Th1, Th2, Th17, regulatory T cells and APCs [18]. Therefore, dual-face γδ T cells are a particularly interesting population during pregnancy, a state where high and stage-dependent plasticity of the maternal immune system is necessary in order to equilibrate between immunosuppression of specific responses towards the fetus and ability to fight infections. In this study, we aimed to examine the proportion, phenotype and TCR γ and δ repertoires of γδ T cells at the MFI and in the blood of pregnant women.

## 2. Results

### 2.1. Increased γδ T-Cell Number at the MFI during Human Early Pregnancy

**Figure 1:**
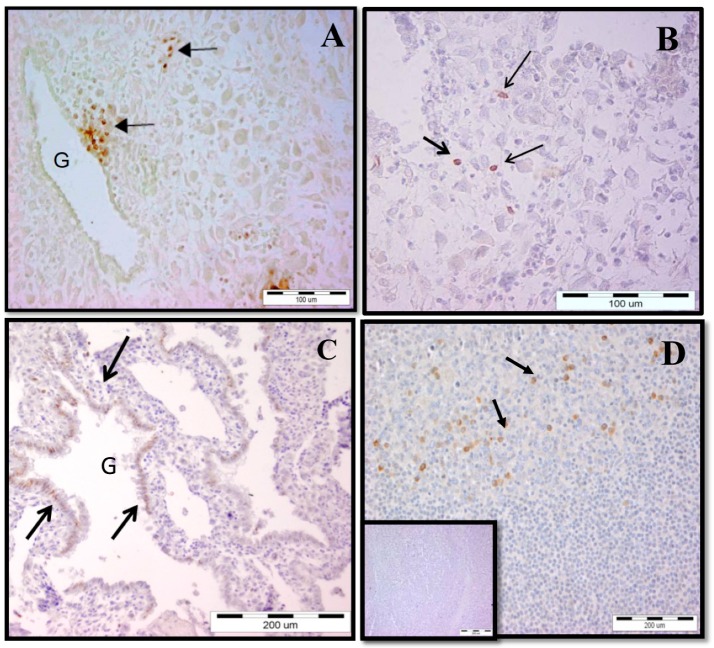
*In situ* visualization of γδ T cells (arrows) at the maternal-fetal interface during early pregnancy. (A) Periglandular clusters of γδ T cells; (B) γδ T cells scattered as single cells in decidual stroma; (C) intraepithelial γδ T cells in decidual glands; (D) staining for γδ T cells in human tonsils (positive control), and an inset is shown as a negative control. G: decidual gland.

**Figure 2:**
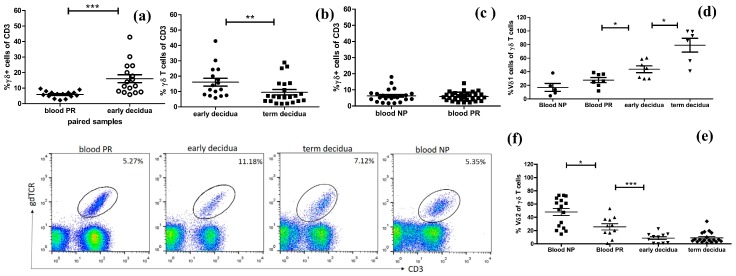
Ex vivo numbers of total γδ T cells and γδ T-cell subsets during pregnancy measured by FACS. (**a**) An increased γδ T-cell number in the decidua compared to that in the blood (early pregnancy, paired samples); (**b**) higher number of γδ T cells in early than in term deciduae and comparable γδ T-cell numbers in the peripheral blood of pregnant (PR) and non-pregnant (NP) women (**c**); (**d**) higher amount of Vδ1 cells in decidual tissues compared to that in the blood of PR women (paired samples) and predominance of this subset in the decidua at term; (**e**) conversely, the pathogen-reactive Vδ2 subset dominated the blood of NP women and decreased in the blood of PR women, at MFI Vδ2 cells were in a lower amount being less than 10% of γδ T cells; (**f**) representative FACS plots showing the number of γδ T cells derived from early and term deciduae and peripheral blood of PR and NP women. The number on the top right corner of each plot denotes the percentage of γδ T cells among CD3+ T cells. Data in the graphs are presented as mean ± s.e., obtained from Mann–Whitney and Wilcoxon matched pairs tests; * *p* < 0.05, ** *p* < 0.01, and *** *p* < 0.001.

We found that γδ T cells are resident cells of decidual tissue showing specific location. During early pregnancy, γδ T cells were distributed in periglandular clusters, intraepithelialy into decidual glands, and scattered as single cells (Figure 1). Being in periglandular clusters, γδ T cells were positioned in close contact with the basolateral portion of the glandular epithelium (Figure 1A). To our knowledge, for the first time, we detected intraepithelial γδ T cells in decidual glands in human pregnancy (Figure 1C). An ex vivo analysis of γδ T lymphocytes obtained from the decidua was undertaken and compared with matched maternal peripheral blood during early pregnancy. We observed three folded frequencies of γδ T cells among all T cells in the decidua than in the blood of pregnant women (16.08 ± 2.55% vs 5.91 ± 0.56%, *p* = 0.0005, *n* = 16, paired samples, Figure 2a). At term delivery, the proportion of γδ T cells (of CD3 T cells) at the MFI decreased significantly as we compared it in early pregnancy decidua with that in the decidua at term (16.08 ± 2.55%, *n* = 16 vs. 9.53 ± 1.73%, *n* = 22, *p* = 0.0097, Figure 2b). No difference in γδ T-cell numbers in the peripheral blood between pregnant and non-pregnant women was detected (5.73 ± 0.43%, *n* = 29 vs. 5.71 ± 0.53%, *n* = 23, *p* = 0.7822, Figure 2с). The number of decidual T cells remained stable over the course of pregnancy and constitutes about 20% of decidual lymphocytes (Figure S1).

### 2.2. Accumulation of γδ T Cells at the MFI Is Restricted to the Vδ1 γδ T-Cell Subset

Next, we determined the proportions of the main subsets of γδ T cells. Although decidua basalis is a region intimately associated with a large volume of maternal blood and in general there would be a likelihood of peripheral blood contamination, our findings showed differential distributions of both Vδ1 and Vδ2 γδ T-cell subsets. As we expected, the decidua was dominated by the Vδ1 subset. During early pregnancy, we found significant increase of Vδ1 subset at the MFI compared to that in the blood of pregnant women (43.64 ± 5% vs. 24.4 ± 3.6%, *n* = 7, *p* = 0.0156) and a predominance of this subset in the decidua at term delivery (79% of all γδ T cells, *p* = 0.0350, Figure 2d). The proportions of Vδ1 within peripheral γδ T cells were comparable between pregnant and non-pregnant women (27.68 ± 3.7% and 16.92 ± 5.85%, respectively, *p* = 0.1490). Conversely, the pathogen-reactive Vδ2 cells dominated the blood of non-pregnant women as compared with pregnant ones (48.07 ± 5.42% vs. 25.62 ± 4.69%, *p* = 0.0191, Figure 2e). At the MFI, this subset was in a relatively lower number during early and term pregnancy being less than 10% of γδ T cells (8.63 ± 2.21% and 9.03 ± 1.9%, respectively, *p* = 0.8973). Vδ2 γδ T cells in the early decidua were three times less than their counterparts in the blood of pregnant women (8.63 ± 2.21% vs. 25.62 ± 4.69%, *p* = 0.0010).

### 2.3. Early Pregnancy Decidua is Enriched with Activated, Terminally Differentiated and Pro-inflammatory γδ T Cells

**Figure 3:**
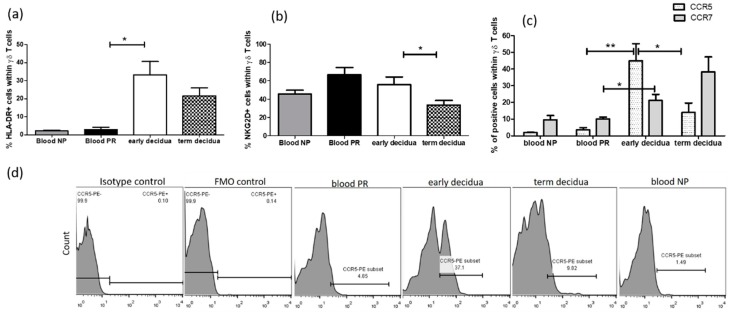
Phenotype of γδ T cells during human pregnancy: (**a**) prevalence of activated (HLA-DR+) γδ T cells at the MFI but not in the blood of both pregnant (PR) and non-pregnant (NP) women; (**b**) more than half of γδ T cells expressed NKG2D regardless of location and γδ+NKG2D+ cells was lowered in amount in the decidua at term; (**c**) expansion of pro-inflammatory γδ T-cell effectors (CCR5+) in early pregnancy decidua where naïve/memory CCR7+γδ+ cells were also in a higher amount compared to the peripheral blood counterparts; (**d**) representative histograms of pro-inflammatory chemokine receptor CCR5 expression on γδ T cells from early and term deciduae and peripheral blood of PR and NP women. The gate is put on CD3+γδ+ cells, FMO: fluorescence minus one control. Graphs show the mean ± s.e., *n* = 5–8, paired and unpaired *t*-tests, GraphPad Prism v.4. * *p* < 0.05, ** *p* < 0.01, *** *p* < 0.001.

Further we performed comparative analysis of the phenotype of γδ T cells during human pregnancy. Interestingly, we found that during early pregnancy, decidual γδ T cells appear to acquire an experienced and differentiated phenotype. A significant proportion of decidual γδ T cells was activated, shown by expression of the late activation marker HLA-DR, which was not the case for their peripheral blood counterparts (36.85 ± 7.92% vs. 1.64 ± 0.22%, *p* = 0.0313, paired samples, Figure 3a). The enriched γδ+HLA-DR+ cells in early gestation were present at term as well (36.85 ± 7.92% and 21.68 ± 4.37%, respectively, *p* = 0.2949). No activated γδ T cells were observed in the blood of neither pregnant nor non-pregnant women (Figure 3a). In line with the assumption that the activating NKR NKG2D is a hallmark receptor for γδ T cells, we revealed highly enriched NKG2D+γδT+ cells, regardless pregnancy status: γδ T cells expressing NKG2D were in high and comparable numbers in both the blood and decidua from the first trimester pregnant women and in the blood of non-pregnant controls and significantly decreased at the MFI at term (Figure 3b).

**Figure 4:**
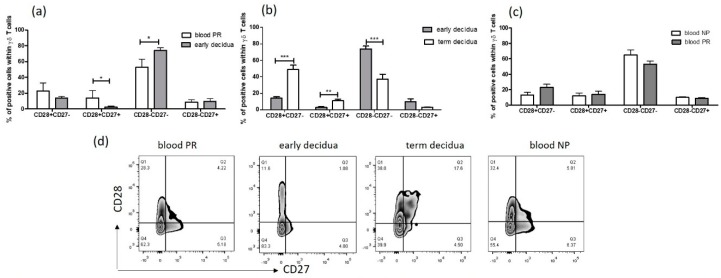
Early pregnancy decidua is dominated by fully differentiated γδ T cells: (**a**) during early gestation, a predominance of fully differentiated effector (CD27−CD28−) γδ T cells in the decidua was found whereas the naïve/memory (CD27+CD28+) γδ T cells were in a negligible amount, in the blood of pregnant women half of γδ T cells were fully differentiated effectors and the remaining γδ T cells were with the naïve/memory and transitional phenotype; (**b**) the early human decidua was dominated by fully differentiated γδ T-cell effectors and no naïve/memory γδ T cells were detected, whereas the term decidua was populated mainly with γδ T cells with the naïve and transitional phenotype; (**c**) the differentiation status of peripheral blood γδ T cells during early pregnancy was similar between non-pregnant (NP) and pregnant (PR) women; (**d**) representative FACS plots show CD27 and CD28 expression for CD3+γδ+-gated cells from early and term deciduae, and from peripheral blood of PR and NP women. Graphs show the mean ± s.e., *n* = 5–6, paired and unpaired *t*-tests, GraphPad Prism v.4. * *p* < 0.05, ** *p* < 0.01, and *** *p* < 0.001.

The advanced or “late” stage of differentiation in conventional T cells is characterized by the downregulation of the costimulatory molecules CD28 and CD27 [19]. These markers have also been used to identify differentiated human γδ T cells [20]. The co-stimulatory molecules CD27 and CD28 can be used as cell surface markers to discriminate naïve and memory T cells (CD27+CD28+) from terminally differentiated effector T cells (CD27−CD28−). CD27+CD28− and CD27−CD28+ subsets are intermediate stages in the differentiation pathway from CD27+CD28+ to CD27−CD28−. By using CD27/CD28 pattern of co-expression, we determined γδ T-cell differentiation status during pregnancy. Strikingly, the dominant population among γδ T cells in the decidua at early pregnancy was that of fully differentiated effector γδ T cells (CD27−CD28−), which comprised 74 ± 3.3% of the whole γδ T-cell population (Figure 4a). Although this subset was well presented in the matched maternal blood, a significant decrease was observed (53 ± 4.1%, *p* = 0.0225, Figure 4a). Following effector γδ T cells at the MFI, we detected a drop in their number at term (37.34 ± 5.8%, *p* = 0.0012, Figure 4b). Interestingly, naïve/memory γδ T cells (CD27+CD28+) showed an inverse pattern of distribution. They were absent from early decidua and found to reach 20% of all γδ T cells in the matched blood of pregnant women (*p* = 0.0087, Figure 4a). At term, naïve/memory γδ T-cell proportion within γδ T cells increased at the MFI compared to that in early decidua (11 ± 2%, *p* = 0.0047, Figure 4b). Note that half of γδ T cells in the term decidua were CD28+CD27− showing transitional differentiation profile. As can be seen in Figure 4c, no difference in the proportions of γδ T-cell effectors and naïve/memory γδ T cells in the blood between pregnant and non-pregnant women was found (γδ T-cell effectors: pregnant (53 ± 4.1%) and non-pregnant (65 ± 6.8%), *p* = 0.0823, and naïve /memory γδ T cells: pregnant (14 ± 3.8%) and non-pregnant (12 ± 3.8%), *p* = 0.7538).

To define the migratory properties of γδ T cells, as deduced by chemokine receptors expression, we checked the expression of homeostatic (lymph node homing) chemokine receptor CCR7 and expression of inflammatory (tissue-homing) chemokine receptor CCR5. CCR7 is a chemokine receptor that functions as a homing receptor in the migration of naïve and memory T cells to secondary lymphoid tissues and serves to define subsets of central memory (CM, CCR7+) and effector memory (EM, CCR7−) T cells [21]. CCR5 has been reported to be preferentially expressed on CCR7neg CD45ROpos Th1 effectors producing IFN-γ [22,23,24]. It appears that CCR7 is gradually lost as T cells differentiate and there is a clear progression starting from CCR7+CCR5− (naïve/memory), to CCR7−CCR5− or CCR7+CCR5+ (transitional stage), and then to CCR7−CCR5+ (effector/memory) T cells [21]. Our results showed distinctive chemokine profile of decidual and peripheral blood γδ T cells in early and late pregnancy. As illustrated in Figure 3c, there was an upregulation of CCR5 on the decidual γδ T cells but not on those in the blood of gravid women (47.33 ± 8.67% vs. 3.20 ± 1.07%, *p* = 0.0027). At term gestation, the number of CCR5+γδ+ T cells at the MFI decreased significantly (13.97 ± 5.64%, *p* = 0.0132). As we expected, a negligible number of CCR5-positive γδ T cells was observed in the peripheral blood, regardless of pregnancy (pregnant 3.20 ± 1.07% and non-pregnant 1.9 ± 0.2%, *p* = 0.3057). As can be seen in Figure 3c, during early pregnancy, the number of decidual CCR7+γδ+ T cells was doubled (21.1 ± 3.7%) compared to that in the matched blood (10.13 ± 1.33%, *p* = 0.0182) and slightly increased at term delivery (38.25 ± 9.02%, *p* = 0.1374). Early pregnancy did not affect the frequency of peripheral blood γδ T cells, expressing CCR7 shown by lack of difference between pregnant and non-pregnant women.

### 2.4. Polyclonal Profiles of All δ and γ Chains and Highly Restricted (Oligoclonal) Repertoire of γ9 Chain of γδ T Cells during Human Pregnancy

**Figure 5:**
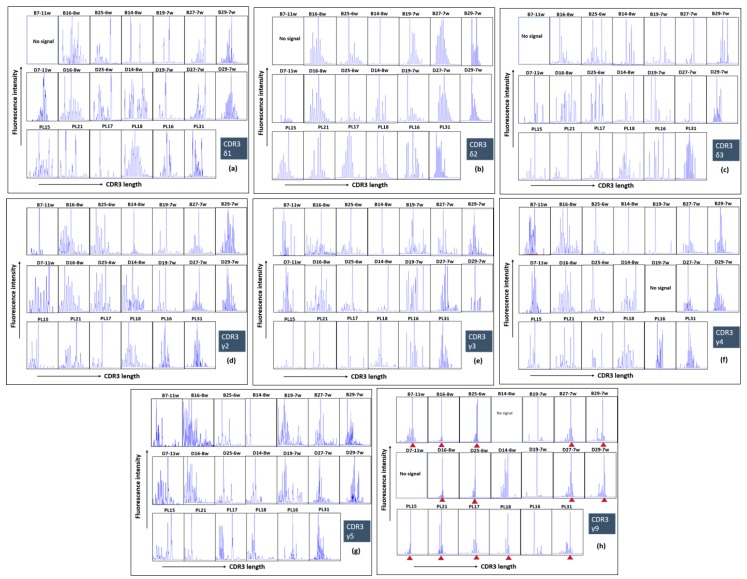
δ1, δ2, δ3 repertoires (**a**–**c**) and γ2, γ3, γ4, γ5 and γ9 repertoires (**d**–**h**) in paired blood and decidua (B-D early pregnancy) and in term decidua (PL) samples. Note predominantly polyclonal repertoires of all δ and all γ chains, whereas CDR3 of γ9 chain was highly restricted with enriched CDR3 regions with one and the same length (**h**, red arrows). Each box represents the spectratyping data of one woman of the indicated CDR3 chain with gestational age in weeks.

Since we found an enrichment and specific phenotype of γδ T cells in the human early pregnancy decidua, we assumed that early pregnancy might impact on their TCR repertoires. As shown in Figure 5, a screening of a complete set of Vδ and Vγ family transcripts could be easily achieved using isolated primary cells, thereby avoiding the bias that can be produced from culture (clones). CDR3 size spectratyping of all δ and γ chains from the decidua and blood of 7 women at early pregnancy (6–11 gw (gestation week)) and from the decidua of 6 term pregnant women (38–40 gw) revealed two main findings: 1) polyclonal CDR3 repertoires of the δ1, δ2 and δ3 chains and γ2, γ3, γ4 and γ5 chains within the samples derived from the blood, early decidua and term decidua and 2) an oligoclonal expansion of Vγ9 transcripts and highly restricted CDR3γ9 repertoire in the vast majority of tested samples with substantial overlap between the compartments—blood, early decidua and term decidua. Note that CDR3 regions with one and the same length persisted throughout three compartments tested—decidua and blood (1st trimester) and in the decidua (3rd trimester). We detected some overlap between the Vδ and Vγ TCR repertoires of decidual γδ T cells and those of circulating γδ T cells; however, different dominant Vδ and Vγ T cell clones were present in the term decidua (Figure 5). Moreover, in the term decidua, the repertoires were more focused, typically resulting from the presence of a relatively small number (e.g., <5) of heavily expanded clonotypes. CDR3 spectratyping of Vδ3 transcripts from the early and term deciduae, and from peripheral blood of healthy pregnant women demonstrated that oligoclonal expansions were present in these three compartments without overlap.

## 3. Discussion

In this study, we demonstrated that γδ T lymphocytes with diverse TCR repertoires can expand, differentiate, and acquire effector phenotypes at the MFI during early human pregnancy, while no significant changes in their counterparts in the blood of pregnant women were observed. Our ex vivo analysis of γδ T cells revealed an enrichment of activated, terminally differentiated pro-inflammatory γδ T-cell effectors at the MFI during early human pregnancy. To characterize γδ T cells, we combined ex vivo FACS for accurate quantitative analysis and IHC to show spatial allocation of γδ T cells in the gestational tissues with evidence for specific cell–cell interactions. We found an increased γδ T-cell number in the early pregnancy decidua in comparison to those in the matched maternal blood and in the term decidua, suggesting possible recruitment and the special role of γδ T cells at the MFI during early human pregnancy. Earlier studies have shown incoherent results about γδ T-cell amount at the MFI in human pregnancy—between 5% and 30% of T cells [13,15,25,26,27] and no difference between the numbers of decidual and peripheral γδ T cells was observed [15]. The sensitivity to fixation of γδ ΤCR and the methodology of cell isolation used might reflect this controversy. We and others have not found any significant fluctuation in γδ T-cell number in the blood between pregnant and non-pregnant women [15,27]. We confirmed that γδ T cells as a part of DALT were localized as dense infiltrates in close proximity to the basal lamina of the decidual glands or scattered as individual cells randomly dispersed between stromal cells [13,28] and extended this finding showing for the first time truly intraepithelial γδ T cells in decidual glands. Intraepithelial γδ T cells have been shown in the murine decidua [29] and in the endometrial epithelium of pregnant sheep uteri, where they may control trophoblast invasion [30]. Intraepithelial location and clustering in the vicinity of the decidual glands have been reported previously for dNK cells [3]. Recently published data showed that decidual glands play a greater role during early pregnancy than previously anticipated by providing growth factors, cytokines and T-cell immunosuppressive factors in addition to nutrients in the intervillous space until at least 10 weeks of gestation [31,32,33]. Moreover, rather than specifically focusing on invasion into spiral arteries, EVTs also invade into uterine glands (endoglandular trophoblast) from the very beginning to open them toward the intervillous space [34]. What is the mechanism of γδ T-cell attraction and what could be the role of γδ T cells gland-associated clusters in the local immune regulation during human pregnancy remain to be elucidated. In line with previous reports on local T-cell maturation in the human decidua, we are tempted to suggest periglandular γδ T-cell clusters as possible sites of γδ T cells differentiation and/or immune modulation. Not unexpectedly, we observed that the vast majority of the human decidual γδ T cells were Vδ1+, while most of peripheral blood γδ lymphocytes expressed Vδ2 TCR [35,36,37,38]. Our finding about the prevalence of Vδ1 subset at the term delivery decidua agreed with the data showing that Vδ1 cells are also the main fraction in cord blood as well [39,40,41]. The major differences in γδ T-cell subsets and the inverse pattern of distribution among decidua and blood of pregnant women may reflect their distinct immunomodulatory functions in both compartments. Like other mucosae, the decidua is the natural habitat of resident Vδ1+γδ T cells where they are involved in the immune surveillance. Mucosa-based Vδ1+ cells have been shown to recognize MHC class I chain-related sequences A and B (MICA and MICB) [42]. These antigens are expressed by normal and transformed EVTs and other cell types of epithelial origin and are modulated by stress, inflammation, infection and cancer [12,42,43,44]. The natural receptor of MICA/B is NKG2D widely expressed on γδ T cells and dNK cells [45,46], and we detected highly increased NKG2D on γδ T cells, regardless of pregnancy status. It seems that this expression is mandatory on both Vδ1 cells [47] and pathogen-reactive Vδ2 cells [48]. Elevated levels of soluble MIC molecules derived from syncytiotrophoblasts and found in pregnancy sera were able to downregulate the NKG2D receptor and to impair the cytotoxic function of PBMC (peripheral blood mononuclear cells) from healthy pregnant women and thus, the novel mechanism of immune evasion of the fetal allograft through fetal MIC and maternal NKG2D interactions was proposed [46]. Since the Vδ1 subset was the dominant cells among γδ T cells in the decidua (50% and 79% of γδ T cell population in early and term deciduae, respectively) versus 20% in the blood of pregnant women, we could rather assume the importance of Vδ1 cells at the MFI than in the blood of pregnant women. However, some studies showed the importance of Vδ1 cells in the blood of pregnant women as well, connecting preferential use of Vδ1 chains by peripheral γδ T cells with Th2 type of response and healthy pregnancy [38,49].

In terms of receptors expression, we revealed that decidual γδ T cells are phenotypically different from peripheral blood γδ T cells and resembled the activated Th1 cohort as most of the decidual lymphocytes [50,51]. Old and recent data described an accumulation of activated CD4+ and CD8+ effector memory cells in decidual tissue compared to those in the peripheral blood in normal pregnancy [51,52,53]. The majority of human decidual CD4+ and CD8+ T cells are antigen-experienced effector cells, whereas naïve T cells can only be detected in low numbers [52,54]. In this vein, a direct comparison between early and term deciduae in our study indicated predominance of more experienced fully differentiated (CD27−CD28−) γδ T cells in the early pregnancy decidua and more naïve γδ T cells (CD27+CD28+) in the term decidua. CD27−CD28− T cells appeared as terminally differentiated and represent the end-stage cells, secreting a range of cytokines and chemokines including pro-inflammatory Th1-like cytokines [19,55]. Recent data revealed that low or lack of CD27 expression on peripheral blood Vδ1 subsets is consistent with a functional effector status [56] and an enrichment of effector CD27lo/neg Vδ1+ T cells in the liver was found [57].

The fact that effector γδ T cells are more numerous in the decidua compared to those in peripheral blood of pregnant women implies that there is some mechanism for their selective recruitment and/or retention in the decidua. Indeed, we observed an upregulation of tissue-homing pro-inflammatory chemokine receptor CCR5 on the decidual γδ T cells during early pregnancy and neither on γδ T cells in the blood of pregnant women, nor on γδ T cells in the term decidua. Expression of CCR5 has been mostly associated with pro-inflammatory Th1 effector memory T cells that home toward sites of inflammation outside the secondary lymphoid organs [21,58]. Although the published data have identified strong CCR5 expression as a selective feature of ex vivo analyzed peripheral blood Vδ2 T cells in healthy donors [22], we could not associate the predominant CCR5+γδ+ population at the MFI during early pregnancy with Vδ2 cells, accounting for below 10% of total γδ T cells. The mutually exclusive expression of CCR5 and CCR7 suggests exclusive homing potential of γδ T cells to the “mildly” inflamed peri-implantation decidual tissue and lymph nodes, respectively. The predominance of γδ T-cell effectors at the MFI during early human pregnancy nicely correlates with higher CCR5 expression, while the predominance of naïve and transitional γδ T cells in the term decidua relates with high CCR7 expression. It is known that CCR5’s cognate ligands MIP-1α/MIP-1β/RANTES are expressed at the MFI during early pregnancy [59,60,61,62]. Moreover, progesterone significantly increased RANTES production, which may be implicated in the local induction of a Th1-type response necessary for successful implantation [63]. Taking together all these data suggests that CCR5 may be responsible for the selective accumulation and retention of γδ T-cell effectors at the implantation site in the gravid uterus. Given the idea about the immunologically sensitive aspect of pregnancy and conceptus rejection, old dogma postulates the relative absence in the decidua and placenta of chemokines, and molecules that attract leukocytes to tissues. However, the unusual recruitment of specific subsets of leukocytes to the uterus during pregnancy shown in many studies suggests the hypothesis about tightly controlled manner of chemokines expression [50]. The statement that inflammatory responses during gestation are strongly associated with negative pregnancy outcomes is outdated [64]. Although early studies have indicated that the cytokine profile at the MFI was skewed towards Th2 [65], a murine study demonstrated that knockout of the Th2 effector cytokines IL-4, IL-5, IL-9, and IL-13 did not necessarily lead to fetal loss [66]. Moreover, it has been recently shown that appropriate generation of pro-inflammatory response is thought to be a prerequisite for successful implantation [67,68] and that the human decidual lymphocytes have a predominant Th1, Th17, and T regulatory profile [69]. Published data showed that effector T cells at the MFI produce a range of Th1 cytokines including TNF-α and IFN-γ, which are crucial for angiogenesis and arterial remodeling during implantation [70,71] and for the control of trophoblast invasion [72,73,74]. Here, we demonstrated that the effector pro-inflammatory phenotype of γδ T cells is compatible with normal human pregnancy.

Although we detected striking differences in γδ T cells phenotype among the decidua and blood of pregnant women and among early and late pregnancy, we were not able to detect enrichment of γδ T cells with specific TCR repertoires in these compartments. Christmas et al. working with γδ T-cell clones from the human decidua and cervix indicated considerable γδ TCR diversity and suggested that γδ T cells localizing in human female reproductive tissues do not express invariant TCR [36]. By using freshly isolated cells, we partly confirmed the results above. We found an expansion of γδ T lymphocytes with diverse CDR3 repertoires of the δ1, δ2 and δ3 chains and γ2, γ3, γ4 and γ5 chains in the decidua and blood of pregnant women. However, we detected an oligoclonal expansion of γ9 transcripts with highly restricted CDR3γ9 repertoire in the vast majority of the investigated samples. Moreover, CDR3γ9 with one and the same length persisted throughout the three compartments tested (decidua and blood from the first trimester, and in the term decidua). In pregnant and non-pregnant mice γδ T cells in vagina, uterus and tongue epithelium express homogeneous TCR with the same V delta 1 chain [75]. It has been shown that the presence of γδ T cells with invariant receptors in murine vagina and uterus is to combat a commonly encountered infectious agent [75,76]. It is uncertain whether this is the exclusive function of such cells in human tissues. Published data suggested that the restricted γ9 repertoire is directed against non-MHC-restricted antigens common to pathogens and stressed self-cells and that special canonical Vγ9Vδ2 T cells recognize non-peptidic phosphorylated antigens [77]. It seems that the oligoclonality of Vγ9 repertoire found in our study is not specific for pregnancy. We have recently proved highly restricted oligoclonal Vγ9 repertoire of fetal phosphoantigen-reactive γδ T cells with the germline-encoded Vγ9-JγP CDR3 sequence [78], which is also highly enriched among all CDR3γ sequences within peripheral blood of adults [79,80]. Most likely, γδ T cells with canonical Vγ9TCR are thymus-independent pool [81]. These Vγ9 cells are not clonal as they are paired with diverse Vδ2 chains [56], and this could be the case in the decidua as shown in our Vδ2 spectratyping plots.

We revealed some overlap between the Vδ and Vγ TCR repertoires of decidual and circulating γδ T cells, suggesting a recirculation of γδ T-cell clones between the blood and decidua of pregnant women. In the term decidua, however, the presence of different dominant Vδ and Vγ T cell clones was observed, suggesting that distinct antigens might be recognized at the MFI in early and late pregnancy. Alternatively, a great deal of the diversity of the CDR3 junctions of the δ chain may confer different affinities of the γδ TCR rather than the ability to recognize different ligands [4].

In summary, in this study, we have made the following new observations: (1) specific intraepithelial distribution and enrichment of γδ T cells in early pregnancy decidua basalis than in the matched blood of pregnant women, (2) prevalence of terminally differentiated and pro-inflammatory γδ T-cell effectors into decidua basalis during early pregnancy, (3) polyclonal CDR3 repertoires of the δ1, δ2 and δ3 chains and γ2, γ3, γ4 and γ5 chains and oligoclonal and highly restricted CDR3γ9 repertoire of γδ T cells within decidua basalis (early and term) and blood of pregnant women. TCRγδ cells are very heterogeneous population comprising of different γδ T-cell subsets with more innate or more adaptive features [82,83] and the tissue residence phenotype [57]. Although our study indicates the decidua residence phenotype of γδ T cells during early pregnancy, investigations in large sample cohorts and differential analysis of decidual γδ T-cell subsets are needed.

The existence of terminally differentiated γδ T-cell effectors at the MFI during early pregnancy shown in our study indicates the presence of antigens that might attract an antigen-specific and/or nonspecific γδ T-cell response. The antigen specificity of γδ T cells and their ability to recognize and respond to fetal antigens is a key question that is yet to be answered. Classical αβ T cells in the mammalian species have not been considered able to recognize trophoblasts because of the atypical expression of MHC class I and class II molecules on human trophoblast cells. EVT expresses MHC class I, specifically the low polymorphic HLA-C and non-classical MHC class I molecules, HLA-G and HLA-E and interaction of these molecules with NK cell inhibitory receptors ILT2 (LIR1), KIRs, and CD94/NKG2A on γδ T cells at the MFI deserves special attention in the context of (un)successful placentation. Accumulated data indicated that function of γδ T cells at the MFI might be regulative, i.e., downregulating other cell populations to prevent anti-fetal immunologic reactions via IL-10 and TGFβ and/or to promote EVT function [26,84,85]. At any rate, decidual γδ T cells as “first line defenders” are just properly positioned and ready to respond to eventual common “stress antigens” as markers of cell infection or transformation. Future studies should focus on the mechanisms involved in the influx, expansion and maturation of γδ T-cell subsets at the MFI over the course of gestation, in order to improve our understanding of their nature and role in human pregnancy.

## 4. Materials and Methods

### 4.1. Study Population and Samples

Healthy pregnant women in early pregnancy, directed to elective pregnancy termination (6–12 gw, *n* = 32) and in term pregnancy, directed to delivery (38–40 gw, *n* = 26) as well as healthy age-matched non-pregnant women (volunteers, control group, *n* = 24) were involved in the study. Pregnancies complicated by clinical evidence of infection, steroid treatment, AIDS, alcohol abuse, and/or drug abuse and immune-associated diseases were excluded. This study was carried out in accordance with the Declaration of Helsinki and was approved by Human Research Ethics Committee at the University Obstetrics and Gynecology Hospital “Maichin Dom” and the Medical University, Sofia, Bulgaria (6 November 2016, DN 03/5). Written informed consent was taken from all subjects for the use of blood and tissue samples. Paired samples blood and decidua from women in early pregnancy, term decidua/placental tissue from women in term pregnancy and blood from non-pregnant women were subjected to investigation. Samples were processed within one hour after blood withdrawal and tissue collection. To exclude the effects of labor or vaginal delivery on the decidual cells, only deciduas from pregnant women delivered by the elective cesarean section prior to the onset of labor were selected.

### 4.2. Mononuclear Cells Isolation (PBMC, DMC)

Blood samples were obtained in heparin anti-coagulated vacutainer tubes (BD Biosciences, San Jose, CA, USA). Peripheral blood mononuclear cells (PBMCs) were isolated from blood samples diluted with PBS (1:2 by the standard Histopaque density gradient centrifugation method (20 min/800× *g*, density: 1.077 g/ml, Sigma–Aldrich). The aliquots of PBMCs were used immediately for FACS or stocked frozen in fetal calf serum (FCS) containing 10% dimethylsulfoxide (DMSO) at −80 °C until the RNA extraction. For early-pregnancy decidual tissue (1st trimester), only decidua basalis connected to villous tissue was used and processed after careful separation from trophoblasts. Third-trimester decidua basalis (term decidua) was dissected from the maternal-facing surface of the basal plate, covered by decidua basalis. To avoid selective cell death or selective loss of surface proteins, mechanical disaggregation rather than enzymatic digestion was used to process the decidual tissue and to isolate the decidual leukocytes. Separation of decidual mononuclear cells (DMCs) from early and term decidual tissues were prepared by mechanical disruption of tissue in sterile PBS (5 g/50 mL) followed by sequential filtrations of resultant suspension with a 100 μm metal sieve and a 60 μm strainer (Becton Diskinson, San Jose, CA, USA), and centrifugation at 1500 rpm for 15 min. The pellet was resuspended in sterile PBS, layered on Histopaque and spun at 800× *g* for 20 min (without break). The mononuclear cells were removed from the interface, washed, assessed for viability with trypan blue exclusion (always achieving a purity greater than 95%) and then were used for FACS staining or were frozen in FCS, containing 10% DMSO at −80 °C until RNA extraction. The yield was usually 0.5 × 10^6^–1 × 10^6^ cells per gram tissue.

### 4.3. Cell Labelling and FACS

Freshly separated untouched PBMCs and DMCs adjusted to 1 × 10^6^ cells per sample were immediately used for surface staining of T cell markers. For subset identification, the suspensions were incubated with the following monoclonal antibodies (mAbs) in different combinations: CD3−FITC (clone UCHT1, ImmunoTools, Friesoythe, Germany), CD3 APC (clone UCHT-1, BD Biosciences, San Jose, CA, USA), γδ TCR-PE (clone F11; BD Biosciences), Vδ1-FITC (clone TS8.2, Thermo Fisher Scientific, Waltham, MA, USA), Vδ2-PerCP (clone B6, BioLegend, San Diego, CA, USA), HLA-DR-APC (clone G46-6, BD Biosciences), NKG2D-APC (clone 1D11, BD Biosciences), CD27-eFluor 450 (clone 0323, Thermo Fisher Scientific), CD28-PE/Cy7 (clone CD28.2, BioLegend), CCR7-PE (clone G043H7, BioLegend), CCR5-PE (clone NP-6G4, Thermo Fisher Scientific). The cells were washed with 3 ml ice-cold FACS buffer (PBS containing 0.1% bovine serum albumin, Sigma-Aldrich, Munich, Germany) and incubated with the mAbs for 20 min at 4 °C in dark. After washing with FACS buffer, the cells were fixed in 300 mL 1% paraformaldehyde (Sigma-Aldrich). Flow cytometric analyses were performed on an FACS Calibur or BD™ LSR II instruments and data were processed using CellQuest Pro v.5.1 software or FCS Express™ Diva v.6 software (BD Biosciences). A real-time gate was set around the viable lymphocytes based on their forward scatter/side scatter profile. Approximately 50,000 cells per sample were acquired for analysis. Compensation controls were prepared simultaneously with sample processing using cells stained with a single mAb. Fluorescence minus one (FMO) and isotype-matched immunoglobulins at the same concentration as the primary mAbs were used as controls for nonspecific immunofluorescence and to set gates. T lymphocytes were selected based on forward and side scatter plots and staining for CD3. Within the CD3+ cell population, γδ T cells were distinguished, and to define Vδ1 or Vδ2 subsets, the gate was set on γδ+CD3+ population. Data acquisition and FACS analysis of all markers are not available on all samples due to limitations on lymphocyte yield of some of the isolates. The exact numbers of samples for each analysis are shown in figure legends.

### 4.4. Immunohistochemistry (IHC)

Early and term pregnancy decidual tissues (10 mm × 10 mm × 10 mm) were fixed in 10% buffered formalin or formalin-free HOPE fixative (Innovative Diagnostik-System, Hamburg, Germany). The samples were routinely processed, embedded in paraffin wax and sectioned at 5 μm. The paraffin sections were proceeded and stained with hematoxylin/eosin for histological investigation and then the selected slides were subjected to IHC for γδ T cells visualization using three-step biotin–streptavidin enzyme method. We have used UltraTek Anti-Polyvalent visualization system following the recommendations of the manufacturer (SkyTec Laboratories, Logan, UT, USA). Briefly, dewaxed and rehydrated sections were incubated with Super block to inhibit non-specific binding. Endogenous peroxidase activity was blocked with 3% hydrogen peroxide in distilled water for 30 min at 37 °C. Primary mouse anti-human TCRγδ mAb (5A6.E9, Thermo Fisher Scientific) diluted in 1% bovine serum albumin/PBS was added for overnight incubation at 4°C in a humidified chamber. After 3 times washing of the slides with PBS, the endogenous biotin was blocked and then incubation with polyvalent biotinylated antibody for 10 min at room temperature was done. After washing 3 times, the slides were incubated with streptavidin-horseradish peroxidase for 10 min at RT. The peroxidase activity was revealed with ready-to-use 3,3-diaminobenzidine tetrahydrochloride (DAB). Nuclei were slightly counterstained with hematoxylin. For negative control, the primary antibody was omitted. Sections from human tonsils proceeded in the same way were used as positive controls for specificity of γδ T cell staining.

### 4.5. Spectratyping

The method which we have used to analyze the TCR repertoires of decidual/peripheral γδ T cells was CDR3 length analysis (CDR3 spectratyping). We performed this analysis in an attempt to identify decidua-based dominant γδ T cell clones reflecting in oligoclonal expansion of certain Vδ or Vγ transcripts. A polyclonal repertoire was characterized by a Gaussian distribution of TCR CDR3 lengths within all δV or γV families. Based on the broad range and frequency distribution of TCR δ or γ CDR3 lengths, the band pattern of a polyclonal TCR repertoire had a bell-curved distribution in which the greatest band intensity was at the median length, with bands containing shorter and longer CDR3 regions being approximately equally distributed in decreasing frequency on either side. Adjacent bands must be separated by a distance corresponding to 3 bp (i.e. one codon). An oligoclonal CDR3 profile was characterized by dominant bands of different lengths which did not follow a bell-curve distribution. Total RNA of freshly 1 ×10^6^–5 × 10^6^ PBMCs and DMCs (derived from the early or term decidua) was extracted using Trizol Reagent (Invitrogen, Waltham, MA, USA) and was purified by RNeasy Mini Kit (Qiagen, Venlo, Netherlands). The RNA concentration was measured using the Nanodrop and cDNA was generated using the First Strand cDNA synthesis kit (Fermentas, Waltham, MA, USA). PCR (40 cycles) was performed with primers (Sigma-Aldrich) directed to Cγ (5′–CAAGAAGACAAAGGTATGTTCCAG–3′) and Vγ2 (5′–GCAAGCACAAGGAASCTTG–3′) or Vγ3 (–GTACTATGACGTCTCCACCG–3′) or Vγ4 (5′–ATGACTCCTACACCTCCAGC–3′) or Vγ5 (5′–CCCAGGAGGTGGAGCTGGAT–3′) or Vγ9 (5′–ATCAACGCTGGCAGTCC–3′) resulting in amplification of the sequences containing the CDR3γ2 or CDR3γ3 or CDR3γ4 or CDR3γ5 or CDR3γ9, respectively. For amplification of sequences containing the CDR3δ1 or CDR3δ2 or CDR3δ3, PCR was performed with Cδ (5′–GTAGAAT–TCCTTCACCAGACAAG–3′) and Vδ1 (5′–CTG TCAACTTCAAGAAAGCAGCGAAATC-3′) or Vδ2 (5′-ATACCGAGAAAAGGACATCTATG-3′) or Vδ3 (5′–GTACCGGATAAGGCCAGATTA–3′) primers (Sigma-Aldrich). Amplification products were labeled in an extension, or runoff, reaction using Cγ-specific FAM fluorescent probe AATAGTGGGCTTGGGGGAAAC and Cδ-specific FAM fluorescent probe ACGGATGGTTTGGTATGAGGCTGA. Fluorescent runoff products and TAMRA fluorescent DNA weight markers were loaded on a sequence gel in an automated sequencer (ProGene, Sofia, Bulgaria). CDR3 sizes and fluorescent intensities were analyzed using the PeakScanner 1.0 software (Applied Biosystems, Foster City, CA, USA).

### 4.6. Statistical Analysis

Statistical analyses were performed with Prism Version 5.0 software (GraphPad Software Inc.).

For comparisons of independent groups, a Student *t*-test or the Mann–Whitney test was performed. For comparisons of matched groups, a paired Student *t*-test or Wilcoxon matched test was performed. In figures, *** *p* < 0.001, ** *p* < 0.01, and * *p* < 0.05.

## Supplementary Materials

Supplementary materials can be found at http://www.mdpi.com/1422-0067/20/3/687/s1.

## Abbreviations

TCR	T cell receptor
MFI	Maternal–fetal interface
DALT	Decidua-associated lymphoid tissue
EVT	Extravillous cytotrophoblast
MHC	Major histocompatibility complex
CDR3	Complementary-determining region 3
NK cells	Natural killer cells
APC	Antigen-presenting cells
mAb	Monoclonal antibody
