# Supplementary material for: Early Pregnancy Human Decidua is Enriched with Activated, Fully Differentiated and Pro-Inflammatory Gamma/Delta T Cells with Diverse TCR Repertoires
Source: Int J Mol Sci. 2019 Feb 5;20(3):687. doi: 10.3390/ijms20030687 (PMC6387174; doi:10.3390/ijms20030687)
Supplement: Supplementary file 1 [file ijms-20-00687-s001.pdf]

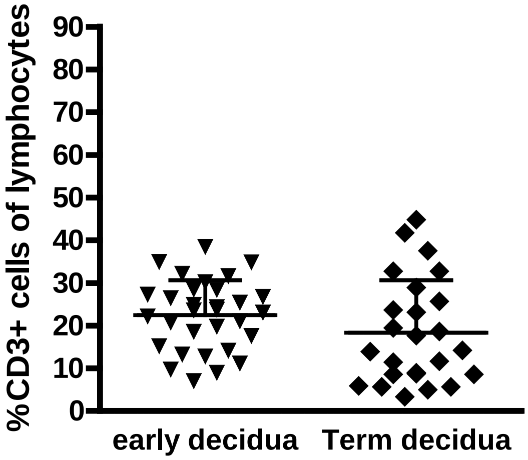

p=0.1389, unpaired t test

The number of decidual T cells remained stable over the course of pregnancy and constitutes about 20% of decidual lymphocytes (unpublished paper, DimovaT).
